# Digital tools of analysis and data integration facilitate synergy between mouse and human brain research and enable translation

**DOI:** 10.1007/s00335-024-10072-1

**Published:** 2024-09-29

**Authors:** Sabine M. Hölter, Lillian Garrett, Sebastian Bludau, Katrin Amunts

**Affiliations:** 1 German Research Center for Environmental Health, Institute of Developmental Genetics and German Mouse Clinic, Helmholtz Munich, Neuherberg, Germany; 2 Technical University Munich, Munich, Germany; 3 DZPG (German Center for Mental Health), Partner Site Munich, Munich, Germany; 4 German Research Center for Environmental Health, Institute of Experimental Genetics and German Mouse Clinic, Helmholtz Munich, Neuherberg, Germany; 5 Research Centre Jülich, Institute of Neuroscience and Medicine (INM-1), 52425 Jülich, Germany; 6 Medical Faculty and University Hospital Düsseldorf, C. & O. Vogt Institute for Brain Research, Heinrich Heine University Düsseldorf, 40225 Düsseldorf, Germany

In the age of precision medicine, which is very much driven by successes in the field of mammalian genetics and genomics, the inclusion of digital approaches from brain research offers new opportunities to the field. Data integration, AI-based analysis as well as modeling and simulation from the molecular level to the level of whole organs or organisms create new impact on the understanding of many human diseases (Amunts et al. 2024). This is particularly—but not only—true in the field of Rare Diseases. According to the Orphanet database, 300 Mio people worldwide live with a rare disease, and it is estimated that 36 million people are affected in the EU. About 72% of rare diseases have a genetic origin, and approximately 70% of rare diseases already start in childhood (Nguengang Wakap et al. 2020). Only limited patient cohorts exist for any given rare disease, which is why genetic animal models may be particularly useful in this area (Silva-Buttkus et al. 2023). We believe that the combination of computational analyses of comprehensive phenotype data of such models with digital tools for brain research will open up new avenues to inform and guide treatment strategies, because many rare diseases affect neurodevelopment, and thus brain function.

Neurodevelopmental disorders (NDDs) may result in multiple permanent brain dysfunctions concerning sensory, motor, emotional, learning and memory abilities, hampering personal wellbeing, quality of life and socioeconomic success. Studies on NDD prevalence rates are mainly available for specific disorders and vary in their methodologies, but a recent systematic review attempting to assess global NDD prevalence as a whole found that (i) multimorbidity was the norm, (ii) prevalence remained stable over time in different cultures, ages, ethnicities, and (iii) differences in sex were consistent, with males being more affected by general psychiatric psychopathology (Frances et al. 2022). These results would have an impact on research strategies and suggest that close cooperation between brain research and the genetics and genomics field is mandatory. They demonstrate the need to study larger cohorts, more complex animal models, and the increasing need to include digital methods such as modeling and deep learning considering that it is impossible to address all the different factors and their interactions experimentally.

Moreover, independent of age or genetic burden, at least one in three people will suffer from a brain disorder in their lifetime (Bassetti et al. 2022; Raggi and Leonardi 2020). These alarming numbers are not only impacting the brain health strategy of the European Academy of Neurology (Bassetti et al. 2022), but also causing MEPs to place brain research at the top of the list of European research priorities (Solis and No 2023). As a result, a European Partnership for Brain Health is in preparation to structure research and innovation in this area (see Home Page CSA BrainHealth—CSA BrainHealth (brainhealth-partnership.eu). And vice versa—embracing genetics and other omics will clearly further advance brain research. A recent review illustrated how in-depth biological studies on rare genetic diseases in model organisms can lead to a deeper understanding of human health in general, including common diseases (Yamamoto et al. 2024). This highlights the importance of cross-species comparisons ensuring that the cellular and molecular mechanisms are conserved in the chosen model organism, because only conserved mechanisms are likely to bridge the gap between rare and common diseases. They also have the potential to become diagnostic or predictive biomarkers.

We recently conducted a cross-species comparison of brain gene expression between mice and humans, focusing on genes that, when knocked out in mice, alter a Schizophrenia-related endophenotype known as prepulse inhibition (PPI). To this end we leveraged the large-scale gene-phenotype resource of the International Mouse Phenotyping Consortium (IMPC) and the region-specific transcriptomic information of the mouse and human Allen Brain atlas (Garrett et al. 2024). The goal was to find overlaps in phenotype and gene expression in relevant brain regions between both species to find genes with conserved functions worthy of further investigation, to better understand genetic contributions to disease-causing neurodevelopmental alterations. As a result, it turned out that the available granularity of regional gene expression data in the Allen Brain Atlas is far better for the mouse than it is for the human.

Here, digital brain research tools can lead us beyond the current state-of-the-art. The pioneering European Human Brain Project has enabled substantial methodological advances such as digital data integration and modelling at multiple scales—from molecules to the whole brain (Amunts et al. 2024). One such advancement was the development of JuGEx (https://www.ebrains.eu/tools/JuGEx), an open, web-based tool for integrating tissue transcriptome and cytoarchitectonic segregation (Bludau et al. 2018). JuGEx combines the analytical benefits of both the Allen Human Brain Atlas regional gene expression data (Hawrylycz et al. 2012) and the three-dimensional cytoarchitectonic maps of the Julich-Brain Atlas (Amunts et al. 2020), allowing for more precision in research in brain regions and disease models regarding gene expression. For example, it is possible to investigate the differential gene expression between two different brain areas, individual volumes-of-interest composed of multiple areas, the entire cerebral cortex, or other three-dimensional anatomical sources. JuGEx proposes maps of the Julich-Brain Atlas, based on reproducible microstructural differences between various brain areas in humans, as three-dimensional search masks to select spatially anchored tissue blocks from Allen Brain. I.e., areas are used as volumes-of interest to select tissue samples analyzed and published as part of the Allen Brain microarray study in the same reference space. In the Allen Brain, over 3000 tissue samples were taken from six different postmortem donor brains, distributed across the entire brain, and the expression levels of over 20,000 genes were determined. The genetic data of the selected samples are then used to identify significant differences in the expression levels of different genes of interest, using statistical methods. The advantage and added value in the digital combination of the data sources from the Julich-Brain Atlas and the Allen Brain microarray study lies in the ability to utilize the Julich-Brain Atlas's information about cytoarchitectonically identified areas and their architectural features such as cell densities and layer thickness, as well as the comprehensive and three-dimensionally anchored data from the Allen Brain Institute. This goes far beyond simple anatomical macro-labels both with respect to microanatomical precision and the underlying information that is linked to the Julich-Brain Atlas.

In a pilot project (see Fig. 1), we applied JuGEx to the list of 29 novel Schizophrenia candidate genes that we discovered in the mouse brain (Garrett et al. 2024): loss-of function of these genes caused a PPI phenotype in mice, and these genes were characterized by neuroanatomical patterning in Schizophrenia-relevant brain regions.

**Fig. 1:**
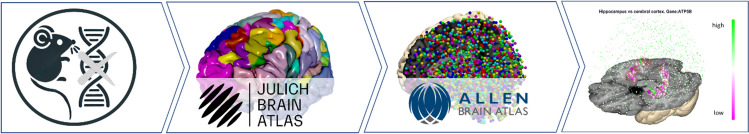
Workflow of applied JuGEx analysis. Twenty-nine Schizophrenia-associated genes were identified in a study using knockout mouse models and the prepulse inhibition phenotype. Brain regions associated with Schizophrenia in humans were identified using the Julich-Brain Atlas. These selected brain regions were used to filter tissue samples from the Allen Brain microarray dataset. The gene expression levels of the area-specific tissue samples were statistically analyzed using JuGEx against the expression levels of all cerebral cortex tissue samples from the Allen Brain dataset

We first focused our analysis on human brain regions in the Julich-Brain Atlas that are homologous to the previously defined rodent prepulse inhibition modulatory circuits (Rohleder et al. 2016). Notably, of the eight brain regions assessed, the most robust differential expression for each of the candidate genes was evident in the hippocampus. Specifically, we found significantly increased expression of the genes *SPOCK1, TPM1, CAMK1, BRD4, FRRS1L, TSPYL2, FAM57B, C1orf96, MIB2* in the hippocampus (containing CA1, CA2, CA3 and DG) compared to the entire cerebral cortex, which was chosen as a broad comparative brain region not specifically associated with Schizophrenia (see Fig. 2 bottom row, red columns). This confirms our previous findings (Garrett et al. 2024) for *CAMK1, BRD4* and *FRRS1L,* which were derived from the human brain analyses, and expands them for *SPOCK1, TPM1, TSPYL2, FAM57B, C1orf96* and *MIB2*, which were derived from the mouse brain analysis, revealing increased expression of these novel Schizophrenia candidate genes in the human hippocampus. Interestingly, the more detailed analysis per hippocampal subregion CA1, CA2, CA3 and DG revealed that the differential gene expression was subregion-specific for some, but not for all genes (see Fig. 2 for details). The hippocampal sub-regions differ not only in cytoarchitecture, but also in their connectivity and molecular fingerprint (Palomero-Gallagher et al. 2020). This finding highlights the importance of the availability of a detailed atlas to be able to detect subregion-specific differences that might be cancelled out if only larger areas are investigated. For example, *RGL1* was significantly upregulated in the CA1 and the CA3 region in comparison to the entire cerebral cortex, and *ARIH1* in CA3 and DG, whereas both genes did not yield a significant differential gene expression in the complete hippocampus analysis (Fig. 2).

**Fig. 2:**
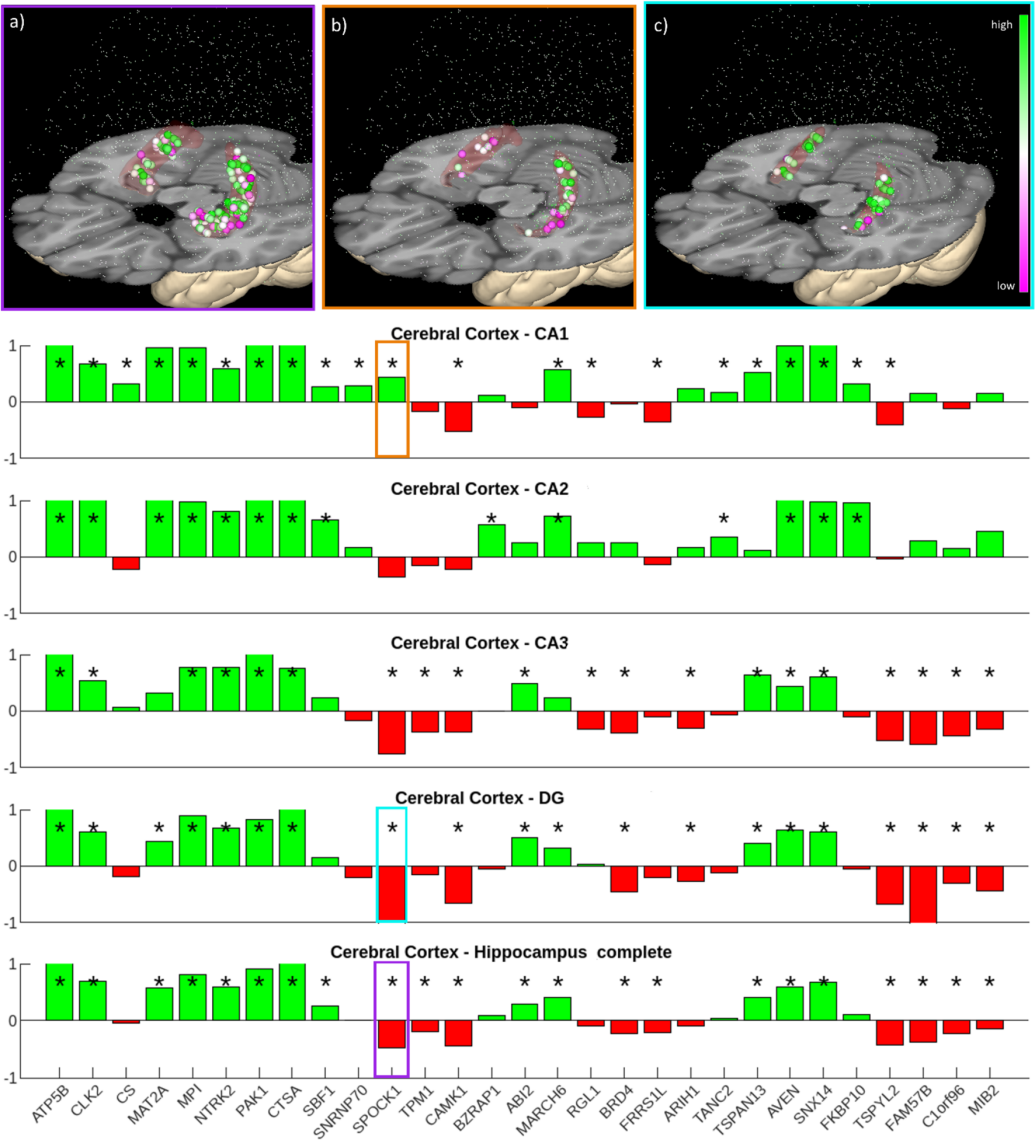
JuGEx analysis of the human hippocampus using the 29 PPI candidate genes discovered in (Garrett et al. 2024). Panels **a**, **b**, and **c** show the positions of the masked tissue samples from the Allen Brain microarray study, with the color of the spheres representing the expression levels of the gene SPOCK1. The colors of the outlines of panels **a**, **b**, and **c** correspond to the frames within the bar plots in the lower part of the figure. Panel a) displays the tissue samples of the entire hippocampus, panel **b** shows the tissue samples exclusively from CA1, and panel **c** illustrates the samples from DG. In the subsequent bar plots, each row represents a JuGEx analysis of all 29 candidate genes in the hippocampal structures: CA1, CA2, CA3, DG, and a combined analysis of all structures (CA1-DG). The analysis compares the expression levels of the investigated genes in these hippocampal regions against the entire cerebral cortex. Red indicates upregulated gene expression in the analyzed structure compared to the whole cerebral cortex, while green indicates downregulated gene expression. Statistically significant differences (p < 0.05, FEW corrected) are marked with an asterisk (*)

A further example underscoring the relevance of detailed granularity of knowledge about brain functions and homologies of brain regions across species, as well as subregion-specificity of analyses is shown in Fig. 3. The bottom row shows the results of our gene list applied to another Schizophrenia-relevant brain region, the dorsolateral Prefrontal Cortex (dlPFC), here represented by the combination of nine distinct Julich-Brain Atlas areas. The involvement of the dlPFC in dysfunctional networks in Schizophrenia is a frequently reported finding. However, the term dlPFC does not encompass an anatomically clearly defined region. Different views on the extent of the dlPFC, e.g. regarding its rostral border, likely contribute to varying findings in this large and heterogeneous part of the human frontal lobe. If the dlPFC is subdivided into area SFS1 and MFG5 (roughly comparable to the historical Brodmann area BA46 (Fig. 3, top row) and SFG2, SFG3, SFS2 and MFG5 (roughly comparable with BA09) (Fig. 3, second row), differences can be seen in the results compared to the combined analysis of all nine dlPFC areas (Fig. 3, third row). This is important because the dlPFC is often seen as an anatomical substrate of functionally very different findings of neuroimaging and physiological studies while these detailed cytoarchitectonic segregations of the region and the differences in expression of each of the genes highlight the heterogeneity of the region and the need to be anatomically precise. Some genes show opposite differential expression results, which cancel each other out if all areas are lumped together—like findings in the hippocampus shown above. This, on the one hand, illustrates the heterogeneity of the dlPFC as a brain region, demanding subregion-specific investigations. On the other hand, it shows that different areas, both discussed as important in Schizophrenia research, can exhibit different gene expression patterns for the same genes. Earlier work of our own group have revealed that the concept of the dlPFC needs to be updated considering the existence of cytoarchitectonic different areas in the inferior frontal sulcus that share cytoarchitectonic and receptorarchitectonic features of both the dlPFC and ventro lateral PFC (Ruland et al. 2022).

**Fig. 3:**
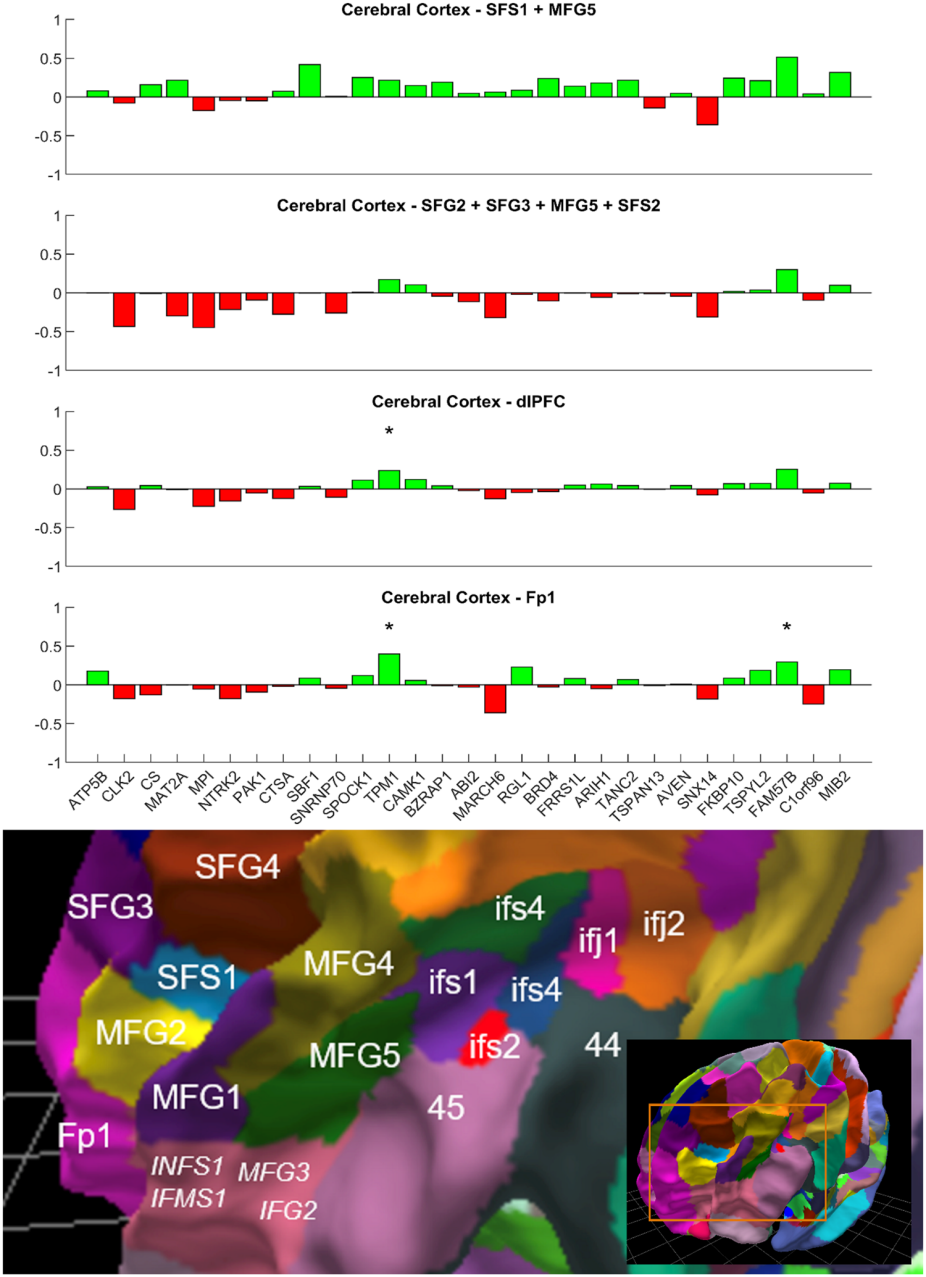
As in Fig. 2, each bar plot represents a JuGEx analysis of all 29 candidate genes. The first row corresponds to the combined structures SFS1 and MFG5 of the dlPFC, and the second row represents the combined structures SFG2, SFG3, MFG5, and SFS2. The third row shows a combined analysis of nine Julich-Brain Atlas areas roughly representing the dlPFC. The last row displays the analysis for area frontopolaris 1 (Fp1), located at the human frontal pole. Red indicates upregulated gene expression in the analyzed structure compared to the whole cerebral cortex, while green indicates downregulated gene expression. Statistically significant differences (p < 0.05, FEW corrected) are marked with an asterisk (*). The lower part of the figure shows an enlarged view of the white-matter surface of the fsaverage brain model labeled with the corresponding Julich-Brain Atlas area names, as well as an overview of the complete surface of the Julich-Brain Atlas. This three-dimensional overview can be interactively explored and utilized in the atlas viewer siibra-explorer of the EBRAINS infrastructure (https://atlases.ebrains.eu/viewer/go/JBA31_whiteM_MPM_frontal_View)

Interestingly, we also found significant differential gene expression for two genes, *TPM1* and *FAM57B*, in the frontal pole, which is a human-specific brain region differing substantially developmentally from the mouse (Fig. 3, bottom row). The frontal pole is often (but not always) interpreted as a region distinct from the dlPFC, while the border between the two does not correspond to an easy-to-define landmark (Bruno et al. 2024). Both of these genes were significantly down-regulated in the human frontal pole in comparison to the cerebral cortex, and *TPM1* was also down-regulated in the dlPFC (Fig. 3, third row). The relevance of this finding is yet unclear, but it is noteworthy that a finding derived from a gene-phenotype-driven analysis of the mouse brain also yields a hit in a human-specific brain region. However, the overall low differential expression of mouse-related PPI genes in the human prefrontal cortex aligns with evidence suggesting greater cross-species overlap in sensorimotor neocortical subdivisions, with less overlap in supramodal regions, such as the frontal pole (Beauchamp, et al. 2022).

It can be that region-specific differences in gene expression are due to variation in cell population proportions. In future analyses it will be important therefore to integrate cell-type specific gene expression information to bring an additional layer of precision. This will be essential to elucidate, for example, the previously unknown function of certain genes. A case in point in our pilot study was the SPOCK1 gene with largely unexplored molecular brain function. We confirmed in humans that it is most highly expressed in the hippocampal CA3 and dentate gyrus subfields (see Fig. 2). Nevertheless, while it predominates in a subset of CA3 pyramidal neurons in the mouse, it is also expressed in endothelial cells and activated astrocytes (Vadasz et al. 2007). Thus, in such instances, the ongoing accrual and availability of single cell datasets (e.g. in the Allen Brain Cell Atlas, https://portal.brain-map.org/atlases-and-data/bkp/abc-atlas) will be invaluable for detecting even more subtle effects, give insights into the cross-species and region-specific gene expression further crystalizing mechanistic interpretations.

Taken together, the results of our pilot digital brain research study show:

(i) that gene-phenotype driven investigation of disease-relevant neuroanatomical patterning in the mouse brain can indeed yield clues that are relevant for the human, in spite of differences in brain development,
(ii) that further in-depth cross-species comparison is necessary to determine which gene and protein functions are conserved enough to translate to the human, and
(iii) that differences in microstructure are linked to area-specific genetic patterns even in regions that are often lumped together, which may blur results or lead to even misleading conclusions.

This marks an important first step opening new possibilities for leveraging large-scale mouse gene-phenotype data alongside human deep phenotyping, neuroimaging and genetic sequencing to identify genetic variants that affect brain development and function. For example, well-characterized, deeply phenotyped human clinical cohorts of Schizophrenia patients could undergo sequencing to assess the candidate genes identified in Garrett et al. (2024) determining their potential role in the disorder. Genes with strong disease associations could then be studied in mouse models exposed to presumed disease triggers, such as early life stress (Senner et al. 2023), followed by thorough assessments of disease-specific deficits in cognitive function, social behaviour abnormalities and brain circuitry changes. This would provide stronger evidence for causality (Ang et al. 2021; Powell and Miyakawa 2006). If successful, these mouse models could be used as valuable tools for testing the efficacy of therapeutic interventions. Of note, since pleiotropy is abundant in both mouse and man (Brown and Lad 2019; Watanabe et al. 2019; Cross-Disorder Group of the Psychiatric Genomics Consortium 2019) and disease classifications in psychiatry still need to be reformed to account for the underlying biology (Kas et al. 2019), it is likely that the genes affecting prepulse inhibition in mice (Garrett et al. 2024) may not only play a role in Schizophrenia, but also in other NDDs.

Such interdisciplinary endeavours as outlined above will clearly be challenging, as it takes time and commitment to understand each other’s methodologies, including their strengths and limitations. Moreover, more digital tools and FAIR data are needed to increase the impact. Nevertheless, we believe this is well worth the effort to advance our understanding of brain disorders, considering that there is “No health without brain health” (Solis 2023). To be goal-directed and to effectively deliver results, this important research needs to be performed within funded projects.

## Data Availability

No datasets were generated or analysed during the current study.

## References

[CR1] Amunts K et al (2020) Julich-Brain: a 3D probabilistic atlas of the human brain’s cytoarchitecture. Science 369(6506):988–992 32732281 10.1126/science.abb4588

[CR2] Amunts K et al (2024) The coming decade of digital brain research: a vision for neuroscience at the intersection of technology and computing. Imaging Neurosci 2:1–35 10.1162/imag_a_00137 PMC12247565 40800542

[CR3] Ang MJ et al (2021) Behavioral tasks evaluating Schizophrenia-like symptoms in animal models: a recent update. Curr Neuropharmacol 19(5):641–664 32798374 10.2174/1570159X18666200814175114 PMC8573744

[CR4] Bassetti CLA et al (2022) The European Academy of Neurology Brain Health Strategy: one brain, one life, one approach. Eur J Neurol 29(9):2559–2566 35538709 10.1111/ene.15391

[CR5] Beauchamp A et al (2022) Whole-brain comparison of rodent and human brains using spatial transcriptomics. Elife. 10.7554/eLife.79418 10.7554/eLife.79418 PMC9708081 36342372

[CR6] Bludau S et al (2018) Integration of transcriptomic and cytoarchitectonic data implicates a role for MAOA and TAC1 in the limbic-cortical network. Brain Struct Funct 223(5):2335–2342 29478144 10.1007/s00429-018-1620-6 PMC5968065

[CR7] Brown SDM, Lad HV (2019) The dark genome and pleiotropy: challenges for precision medicine. Mamm Genome 30(7–8):212–216 31444567 10.1007/s00335-019-09813-4 PMC6759675

[CR8] Bruno A et al (2024) New organizational principles and 3D cytoarchitectonic maps of the dorsolateral prefrontal cortex in the human brain. Front Neuroimaging 3:1339244 38455685 10.3389/fnimg.2024.1339244 PMC10917992

[CR9] Cross-Disorder Group of the Psychiatric Genomics Consortium, Electronic address, p.m.h.e., C. Cross-Disorder Group of the Psychiatric Genomics (2019) Genomic relationships, novel loci, and pleiotropic mechanisms across eight psychiatric disorders. Cell 179(7):1469–148211 31835028 10.1016/j.cell.2019.11.020 PMC7077032

[CR10] da Silva-Buttkus P et al (2023) Knockout mouse models as a resource for the study of rare diseases. Mamm Genome 34(2):244–261 37160609 10.1007/s00335-023-09986-z PMC10290595

[CR11] Frances L et al (2022) Current state of knowledge on the prevalence of neurodevelopmental disorders in childhood according to the DSM-5: a systematic review in accordance with the PRISMA criteria. Child Adolesc Psychiatry Ment Health 16(1):27 35361232 10.1186/s13034-022-00462-1 PMC8973738

[CR12] Garrett L et al (2024) Co-expression of prepulse inhibition and schizophrenia genes in the mouse and human brain. Neurosci Appl 3:104075 10.1016/j.nsa.2024.104075 PMC12244170 40656105

[CR13] Hawrylycz MJ et al (2012) An anatomically comprehensive atlas of the adult human brain transcriptome. Nature 489(7416):391–399 22996553 10.1038/nature11405 PMC4243026

[CR14] Kas MJ et al (2019) A quantitative approach to neuropsychiatry: the why and the how. Neurosci Biobehav Rev 97:3–9 29246661 10.1016/j.neubiorev.2017.12.008

[CR15] NguengangWakap S et al (2020) Estimating cumulative point prevalence of rare diseases: analysis of the Orphanet database. Eur J Hum Genet 28(2):165–173 31527858 10.1038/s41431-019-0508-0 PMC6974615

[CR16] Palomero-Gallagher N et al (2020) Multimodal mapping and analysis of the cyto- and receptorarchitecture of the human hippocampus. Brain Struct Funct 225(3):881–907 31955294 10.1007/s00429-019-02022-4 PMC7166210

[CR17] Powell CM, Miyakawa T (2006) Schizophrenia-relevant behavioral testing in rodent models: a uniquely human disorder? Biol Psychiatry 59(12):1198–1207 16797265 10.1016/j.biopsych.2006.05.008 PMC3928106

[CR18] Raggi A, Leonardi M (2020) Burden of brain disorders in Europe in 2017 and comparison with other non-communicable disease groups. J Neurol Neurosurg Psychiatry 91(1):104–105 31208991 10.1136/jnnp-2019-320466

[CR19] Rohleder C et al (2016) The functional networks of prepulse inhibition: neuronal connectivity analysis based on FDG-PET in awake and unrestrained rats. Front Behav Neurosci 10:148 27493627 10.3389/fnbeh.2016.00148 PMC4954847

[CR20] Ruland SH et al (2022) The inferior frontal sulcus: Cortical segregation, molecular architecture and function. Cortex 153:235–256 35568575 10.1016/j.cortex.2022.03.019

[CR21] Senner F et al (2023) Association of early life stress and cognitive performance in patients with schizophrenia and healthy controls. Schizophr Res Cogn 32:100280 36846489 10.1016/j.scog.2023.100280 PMC9945796

[CR22] Solis S (2023) No health without brain health. In: The Parliament Magazine, see: https://www.theparliamentmagazine.eu/partner/article/no-health-without-brain-health Accessed 6 Dec 2023

[CR23] Vadasz C et al (2007) Mesencephalic dopamine neuron number and tyrosine hydroxylase content: genetic control and candidate genes. Neuroscience 149(3):561–572 17920205 10.1016/j.neuroscience.2007.06.049 PMC2128036

[CR24] Watanabe K et al (2019) A global overview of pleiotropy and genetic architecture in complex traits. Nat Genet 51(9):1339–1348 31427789 10.1038/s41588-019-0481-0

[CR25] Yamamoto S et al (2024) Integrating non-mammalian model organisms in the diagnosis of rare genetic diseases in humans. Nat Rev Genet 25(1):46–60 37491400 10.1038/s41576-023-00633-6

